# Inorganic and Organic Losses of Nitrogen from Upland Regions of Britain: Concentrations and Fluxes

**DOI:** 10.1100/tsw.2001.292

**Published:** 2001-11-20

**Authors:** P. J. Chapman, A. C. Edwards

**Affiliations:** School of Geography, University of Leeds, UK

**Keywords:** nitrate, ammonium, dissolved organic nitrogen, upland streams

## Abstract

The nitrogen (N) composition of streams draining eight upland regions of Britain was compared using monthly samples collected between April 1997 and April 1998. Stream samples were analysed for total N (TN), particulate N (PN), nitrate (NO3), ammonium (NH4), and dissolved organic nitrogen (DON). Concentrations of TN were small, generally less than 1.5 mg N l(-1), were dominated by dissolved forms of N, and varied significantly between regions. NO3 accounted for the majority of variability. Concentrations of DON also varied between regions but to a smaller extent than those of NO3. There were considerable variations in TN fluxes between upland regions, which ranged between 3.8 and 16.1 kg N ha(-1) year(-1). The majority of the variation was due to NO3 fluxes, which were largest in regions receiving largest inputs of atmospheric N deposition and ranged between 1.4 and 13.5 kg N ha(-1) year(-1). Fluxes of DON ranged between 1 and 3.5 kg N ha(-1) year(-1), while fluxes of PN were generally less than 0.5 kg N ha(-1) year(-1), and NH4 fluxes ranged between 0.1 and 0.4 kg N ha(-1) year(-1). NO3 was the dominant fraction (47–84%) of N exported from all upland regions except the Highlands, where DON accounted for 52% of the TN flux. This study has shown that the DON fraction is an important component of the total N transported by upland streams in Britain.

### INTRODUCTION

Over the last decade, studies in Britain have indicated that increased atmospheric nitrogen (N) deposition has led to elevated nitrate ($NO_3$) concentrations in upland streams and lakes[1]. Generally, variation in N deposition only explains about 50% of the variance in surface water $NO_3$ concentrations[1]. Other factors, such as climate, geology, topography, soil type, and land use, have all been shown to be important in controlling $NO_3$ concentrations in surface waters. For example, two catchments of similar size and land use and receiving similar inputs of atmospheric deposition have been observed to vary significantly in the $NO_3$ concentrations of their surface waters, due to differences in their soil types and altitude[2]. Thus variability in stream-water $NO_3$ concentrations may reflect atmospheric deposition patterns, but they are also likely to reflect an effect of geographical variability of physiographic factors. Thus the impact of atmospheric N deposition on the $NO_3$ content of surface waters is likely to vary between catchments/regions with contrasting geology, dominant soil type, vegetation, and climate. Although spatial and temporal patterns in stream $NO_3$ concentrations have been studied in detail within the Welsh uplands[3], considerably less information exists on the variability and behaviour of stream-water $NO_3$ within and between other upland regions of Britain. An increase in $NO_3$ concentrations can lead to the acidification and/or changes in the trophic status of streams, rivers, and lakes. It is therefore important to understand the controls on streamwater N in these seminatural temperate ecosystems, particularly if we want to predict the long-term consequences of increased N deposition on the aquatic environment.

Inorganic forms of N are not the only forms of N present in either soil waters[4] or surface waters[5]. A large number of N-containing organic compounds are also present which are collectively referred to as dissolved organic N (DON). In general, little consideration has been given to quantifying the amount of DON in surface waters in Britain and determining the contribution it makes to total N (TN) concentrations and fluxes in streams and rivers. Without this data it is impossible to evaluate the impact of increased atmospheric deposition on the export of TN from upland catchments. In addition, the impact of DON on the functioning of freshwater ecosystems has largely been ignored. The present study, therefore, was undertaken (1) to investigate the spatial distribution of N forms and concentrations in upland streams of Britain; (2) to quantify the relative contribution made by particulate, organic, and inorganic forms of N to TN concentrations; and (3) to estimate fluxes of particulate, organic, and inorganic forms of N in upland rivers of Britain and to examine spatial variation in these fluxes.

### Uplands of Britain

In Britain, upland and marginal upland landscapes represent 37% of the total land area[6] and are located predominantly in the North and West. The climate is cool and wet, with annual rainfall ranging between 1000 and 3000 mm. The dominant soils include brown earths, podzols, gleys, and peats. On level summits and shallow slopes, where drainage is poor, peat and gley soils occur, while podzolic soils and brown earths have developed on the steeper slopes. These soils are generally acidic, intensely leached, organic-rich, and nutrient-poor, although they contain substantial amounts of organic carbon and nitrogen in the upper horizons. Consequently waters draining these areas generally contain small concentrations of nutrients. Vegetation is dominated by heather (*Calluna*) and acid-grasses (*nardus & festuca*), which are largely sustained by low-intensity sheep and deer grazing. However, agricultural improvement of upland areas has occurred for many years. The methods of improvement have varied but generally have involved a combination of drainage and the addition of lime and fertilisers. Current land-use policy, however, does not favour the continued improvement of upland areas. Over the last 60 years, the major land-use change in upland areas of Britain has been the conversion of seminatural vegetation to plantation forest dominated by Sitka spruce (*Picea sitchensis*).

## METHODS

### Sampling Sites

The climate, vegetation cover, geology, soil type, and other characteristics of the uplands regions vary markedly within Britain. Catchments, therefore, were selected (1) to include a wide range of upland regions, and (2) to cover a wide range of total atmospheric N deposition. Criteria for selection of the catchments were: (1) land use had to be dominated by seminatural vegetation, (2) the river had to be flow-gauged, and (3) the river had to be routinely monitored for water quality by the Environment Agency and the Scottish Environment Protection Agency. In total, 54 catchments in 8 different upland regions were included in the study, the physical characteristics of which are presented in Table 1.

Catchment boundaries were digitised from 1:50,000 Ordnance Survey maps. Using the catchment boundaries, the percentage of different land cover within each catchment was obtained from the Land Cover of Scotland 1988 and Land Cover Map of Great Britain 25-m-resolution databases. The percent-

**TABLE 1**
**Characteristics of the Study Catchments in the Different Upland Regions of Britain**

| Region[a] | Number of Catchments | Total Area of Catchments ($km^2$) | % Land | | Average | | N Input[c] ($kg\ ha^{-1}\ year^{-1}$) |
|---|---|---|---|---|---|---|---|
| | | | >300 m | >600 m | P[b] (mm) | Q[b] (mm) | |
| 1. SW England | 5 | 148 | 62 | 0 | 1980 | 1562 | 10–20 |
| 2. S Wales | 5 | 318 | 63 | 8 | 1604 | 1190 | 20–30 |
| 3. N Wales | 8 | 1478 | 55 | 2 | 1887 | 1568 | 25–30 |
| 4. N Pennines | 10 | 1709 | 51 | 7 | 1127 | 707 | 15–20 |
| 5. Tweed | 5 | 665 | 71 | 7 | 1616 | 1235 | 20–25 |
| 6. SW Scotland | 5 | 553 | 28 | 1 | 1730 | 1338 | 20–25 |
| 7. Cairngorms | 8 | 1247 | 53 | 35 | 1123 | 813 | 10–15 |
| 8. Highlands | 8 | 1347 | 36 | 6 | 1844 | 1376 | 10–15 |

[a] See Fig. 2 for location of regions.

[b] Mean rainfall (P) and runoff (Q) for catchments in each upland region for the period 1991–995 (Σ(rainfall or runoff x catchment area)/Σcatchment area)[8].

[c] Total (wet, dry, and occult) inorganic N deposition ($kg\ N\ ha^{-1}\ year^{-1}$), 1989–1992[7].

age of different soil types within each catchment was derived from the 1:250,000 Soil Survey of Scotland and the 1:250,000 Soil Map of England and Wales. Data for other attributes, obtained from national data sets, included N deposition[7] and catchment rainfall and runoff[8].

### Sample Collection and Chemical Analysis

Between April 1997 and April 1998 inclusive, monthly samples of river water were collected in 1-l polyethylene bottles from all catchments except those in the Tweed and the northern Pennine regions, where samples were collected bimonthly. On return to the laboratory, a subsample was taken for TN analysis and the remainder of the sample was filtered through prewashed, 0.45-µm-membrane filters. $NO_3$–N and $NH_4$–N were determined colorimetrically using a Technicon TRAACS autoanalyser. The detection limit for both $NO_3$–N and $NH_4$–N was 0.01 mg N $l^{-1}$. Total dissolved N (TDN) and TN were determined as $NO_3$–N after oxidation with alkaline potassium persulphate[9]. DON and particulate N (PN) were calculated by difference: DON = TDN – ($NO_3$–N + $NH_4$–N) and PN = TN – TDN.

### Flux Calculations

Interpolation methods are generally used to estimate river fluxes where concentration and flow data are relatively infrequent, as in this study. Of the five commonly used algorithms[10], "Method 5" (Eq. 1) is recommended by the Paris Commission for estimating river fluxes.

$$\text{Flux} = K \times Q_r \times \sum_{i=1}^{n} \left(C_i Q_i\right) \Big/ \sum_{i=1}^{n} Q_r \qquad (1)$$

where K is a conversion factor accounting for the time period of flux estimation and the units, $C_i$ is the instantaneous concentration associated with individual samples, $Q_i$ represents the stream flow corresponding to a sample taken on day i (either instantaneous or daily mean), $Q_r$ is the mean discharge for the period of study, and n is the number of samples. $Q_r$ was derived from a "continuous" flow record, and the mean daily discharge for the day of sampling was used for $Q_i$.

The annual PN, $NO_3$, $NH_4$, and DON fluxes at each site were calculated using Eq. 1 and expressed as the total amount of N exported from each catchment (kg per year). Within each upland region, flux estimates for each catchment were summed and divided by the total area of the catchments within each region. Thus, the resulting PN, $NO_3$, $NH_4$, and DON flux for each region were expressed as the export of N per hectare per year (kg N $ha^{-1}$ $year^{-1}$).

Within each region a small number of stream samples were collected during storm flow conditions. Therefore, to compare fluxes between regions under similar flow conditions, the annual flux at each site was also calculated, after first excluding any samples collected during storms. The discharge thresholds used to identify and exclude such samples were taken as any discharge that exceeded double the mean monthly flow. These "nonstorm" fluxes were then compared with the "total" flux estimates made using all samples.

Producing accurate estimates of chemical loads in rivers is problematic even where regular chemical sampling and continuous flow monitoring are carried out[11]. Since the N data presented in this study are spot samples collected at monthly intervals, sampling tends to occur mostly during baseflow. Samples taken during storms, when changes in N concentration are likely to be greatest, are less likely to be represented in the data set. Therefore, the N fluxes presented in Table 4 are only intended to provide a first approximation and are likely to underestimate the actual load. However, in the absence of more detailed approaches, these estimates still provide an initial insight into variations in PN, $NO_3$, $NH_4$, and DON fluxes from upland regions of Britain.

### Statistical Analysis

One-way ANOVA was used to determine the influence of region on N concentrations in upland streams. The data were skewed and therefore log transformed prior to analysis. Unless otherwise stated, levels of statistical significance were for $p < 0.01$. Relationships between stream-water N forms and catchment attributes were examined by correlation analysis using flow-weighted mean concentrations for the 54 catchments.

## RESULTS

### N Form and Concentration

A significant difference in TN concentrations was observed between upland regions (Table 2). Concentrations were smallest (flow-weighted mean = 0.290 mg $l^{-1}$) in river samples collected from the Highland region, while largest concentrations were observed in streams draining the upland region of the northern Pennines (flow-weighted mean = 1.33 mg $l^{-1}$). The range and variability in $NO_3$–N concentrations in stream samples collected from the catchments within the eight upland regions of Britain are shown in Fig. 1a. $NO_3$ concentrations varied significantly between regions. Concentrations were smallest and least variable in streams draining the Highland region. Low ranges and mean concentrations were also observed in the Cairngorm, Pennine, and southwestern England regions. In contrast, concentrations of $NO_3$–N were generally larger and more variable in stream samples collected from the remaining regions. Largest concentrations of $NO_3$–N were observed in streams draining the upland region of southern Wales. The mean flow-weighted concentration of $NO_3$–N in southern Wales was just over 10 times greater than that in the Highlands (Table 2). Dissolved organic N concentrations also displayed significant differences between regions (Fig. 1b). Smallest concentrations were observed in streams draining northern Wales and the Cairngorms, while largest concentrations were observed in streams draining the Pennine and the southwestern Scotland regions. However, the variation in concentration between regions was less for DON than for $NO_3$; the mean flow-weighted DON concentration was only 3.6 times greater in the Pennines than in northern Wales. Concentrations of both $NH_4$–N and PN were small in all stream samples (generally <0.05 and <0.1 mg N $l^{-1}$, respectively) and displayed little differences between the upland regions of Britain (Figs. 1c and 1d), although the Tweed region had significantly lower $NH_4$ concentrations than did the other upland regions.

**TABLE 2**
**The Annual Flow-Weighted Mean Concentration (mg $l^{-1}$) of N Forms in Streams Draining Each Upland Region**

| Region | TN | PN | $NO_3$–N | $NH_4$–N | DON |
|---|---|---|---|---|---|
| SW England | 0.716 | 0.038 (4) | 0.527 (68) | 0.021 (3) | 0.136 (25) |
| S Wales | 1.19 | 0.067 (3) | 0.958 (84) | 0.016 (1) | 0.181 (12) |
| N Wales | 1.06 | 0.036 (2) | 0.881 (78) | 0.021 (3) | 0.127 (17) |
| Pennines | 1.33 | 0.210 (4) | 0.607 (47) | 0.034 (4) | 0.486 (45) |
| Tweed | 0.756 | 0.039 (2) | 0.447 (61) | 0.013 (2) | 0.264 (35) |
| SW Scotland | 0.833 | 0.021 (3) | 0.512 (57) | 0.030 (3) | 0.281 (37) |
| Cairngorms | 0.388 | 0.018 (5) | 0.228 (57) | 0.024 (6) | 0.134 (33) |
| Highlands | 0.290 | 0.019 (8) | 0.089 (28) | 0.016 (6) | 0.169 (57) |

*Note:* Values in parentheses indicate the percentage contribution each N fraction makes to the flow-weighted mean TN concentration.

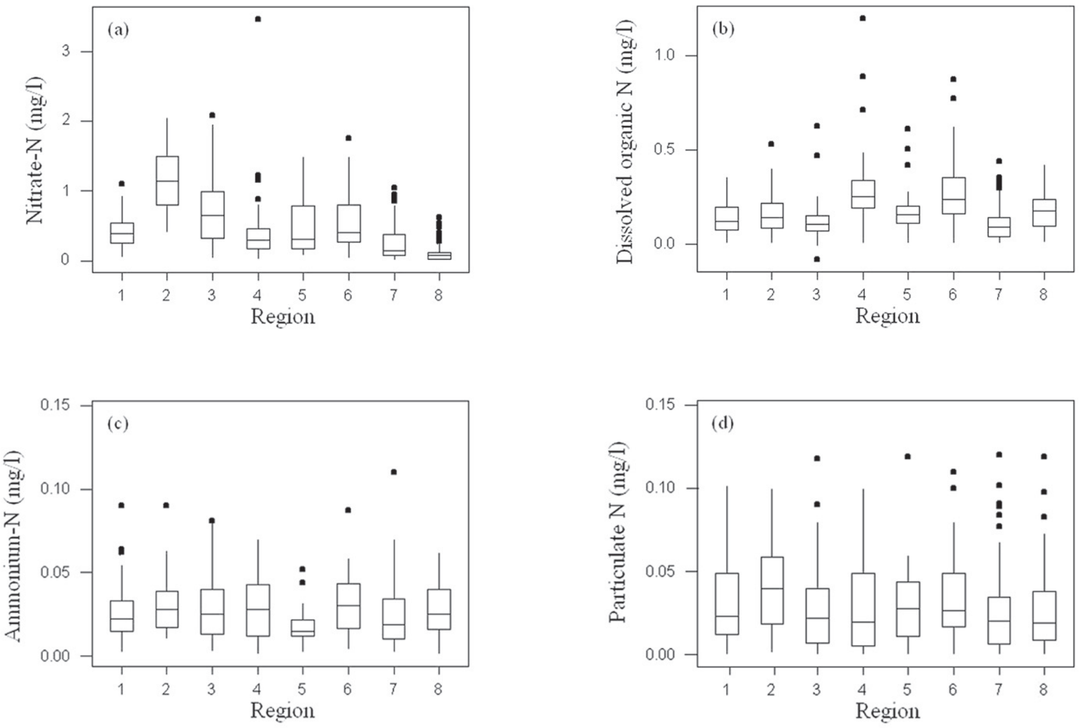

**FIGURE 1.** Box and whisker plots summarising the concentration of (a) nitrate–N, (b) dissolved organic N, (c) ammonium–N, and (d) particulate N in samples of stream water collected from different regions of Britain. Regions are defined in Table 1 and Fig. 2. The middle horizontal line of the box represents the median value. Fifty percent of the data points lie within the box. The ends of each box delineate the upper and lower quartiles. The whiskers show the spread of data and closed circles represent outliers.

The relative contribution of individual N fractions to TN concentrations varied significantly between regions (Table 2 and Fig. 2a). The contribution of PN to TN varied from a minimum of 2% in northern Wales to a maximum of 8% in the Highlands. Thus, TN was dominated by dissolved forms of N in all regions. In the Highlands and Pennines, DON was the dominant fraction, representing 57% and 45% of TN, respectively, while in the other regions $NO_3$ was the dominant form of N. The proportion of $NO_3$ varied from 28% in the Highlands to 84% in southern Wales. $NH_4$ contributed less than 6% to TN in each region. Despite differences in concentrations of both $NO_3$ and DON in streams draining the upland regions of the Cairngorms, southwestern Scotland, and the Tweed, the annual average composition of TN was very similar for these regions.

### N Fluxes

Fluxes of TN during the study period ranged from 3.83 kg $ha^{-1}$ $year^{-1}$ in the Highlands to 16.1 kg $ha^{-1}$ $year^{-1}$ in northern Wales,

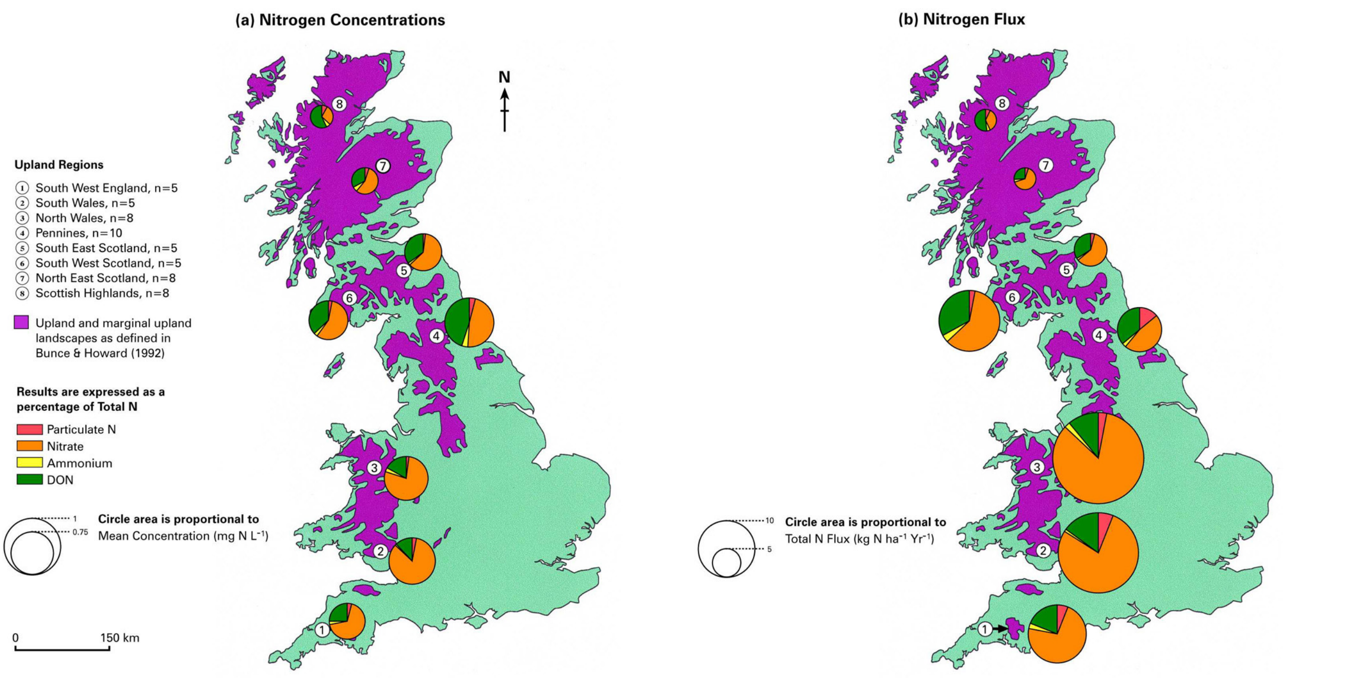

**FIGURE 2.** Map showing the relative contribution of N fractions to the (a) flow-weighted mean total nitrogen concentration and (b) annual total nitrogen flux in streams draining different upland regions of Britain (n = number of catchments in each region).

and there were significant variations in fluxes between upland regions (Table 3). In the Tweed and Pennine regions, TN fluxes were double those observed in the Highlands and Cairngorms (*c.* 3.8 kg $ha^{-1}$ $year^{-1}$), while TN fluxes ranged between 10 and 13 kg $ha^{-1}$ $year^{-1}$ in southwestern England, southern Wales, and southwestern Scotland and were largest in northern Wales. Only a small proportion of TN was exported as PN from the upland regions (3–14%), and fluxes ranged from 0.18 to 1.1 kg $ha^{-1}$ $year^{-1}$. Although small in all regions, PN fluxes were larger (>0.53 kg N $ha^{-1}$ $year^{-1}$) from upland regions in England and Wales than in Scotland, where fluxes were <0.3 kg N $ha^{-1}$ $year^{-1}$ (Table 3).

$NO_3$ fluxes varied from 1.37 kg N $ha^{-1}$ $year^{-1}$ in the Highlands to 13.5 kg N $ha^{-1}$ $year^{-1}$ in northern Wales, and the variations in fluxes between regions were similar to those observed for TN (Table 3). The $NO_3$ fraction accounted for >50% of the TN flux in all regions except the Highlands and Pennines (Fig. 2b). $NH_4$ fluxes were in the range of 0.13 to 0.4 kg N $ha^{-1}$ $year^{-1}$ and accounted for only 1–5% of the TN exported from all the upland regions (Table 3 and Fig. 2b).

Fluxes of DON varied significantly between regions and ranged from 0.99 kg N $ha^{-1}$ $year^{-1}$ in the Cairngorms to 3.54 kg N $ha^{-1}$ $year^{-1}$ in southwestern Scotland (Table 3). However, as observed for DON concentrations, the variability in DON fluxes between regions was less than that observed for $NO_3$ fluxes. The DON fraction represented between 11 and 52% of the TN flux. However, the contribution of DON to the TN flux increased from the south to the north of Britain (Fig. 2b). In southwestern England, southern Wales, and northern Wales the DON contribution was greater than 10% but less than 20%; in the Pennine, Tweed, southwestern Scotland, and Cairngorm regions it ranged between 26 and 36%; and in the Highlands it represented 52% of the TN flux.

In general, TN flux estimates were reduced when samples taken during storm flow conditions were excluded from the flux calculations (Table 3). In the Highland and Cairngorm regions, the flux estimates for all N fractions varied little between the nonstorm and the 'total' fluxes. In all other regions, the nonstorm fluxes of both PN and DON were smaller than the 'total' fluxes. This suggests that both PN and DON consistently increase during storm events in upland streams. In addition, the differences in fluxes of DON between regions were less pronounced; excluding the Cairngorm and southwestern Scotland regions, nonstorm fluxes of DON from the upland regions only ranged between 1.5 and 2.09 kg N $ha^{-1}$ $year^{-1}$. The $NO_3$ flux was smaller in some regions (northern Wales and the Pennines) and larger in others (Tweed, southwestern Scotland, southern Wales, and southwestern England) when samples taken during storm flow conditions were excluded. This suggests that both increases and decreases in stream-water $NO_3$ concentrations occur during storms. However, the regional pattern in nonstorm fluxes of $NO_3$ was little changed from that exhibited by 'total' $NO_3$ fluxes. There was little difference in 'total' and nonstorm flux estimates for $NH_4$ in all regions.

### Relationship between N Flux and Catchment Characteristics

Relationships between fluxes of PN, $NO_3$, $NH_4$, and DON with catchment attributes such as dominant soil types, N deposition,

**TABLE 3**
**The Annual Fluxes of N Forms (kg N $ha^{-1}$ $year^{-1}$) from Streams Draining Each Upland Region**

| Region | No. Storm Samples | TN | PN | $NO_3$-N | $NH_4$-N | DON |
|---|---|---|---|---|---|---|
| **Total Flux** | | | | | | |
| SW England | | 10.25 | 0.55 (5.4) | 7.41 (72.3) | 0.31 (3.0) | 1.98 (19.3) |
| S Wales | | 14.18 | 0.77 (5.4) | 11.10 (78.3) | 0.18 (1.3) | 2.13 (15.0) |
| N Wales | | 16.10 | 0.53 (3.3) | 13.50 (83.8) | 0.29 (1.8) | 1.78 (11.1) |
| Pennines | | 7.81 | 1.10 (14.1) | 3.68 (47.1) | 0.20 (2.6) | 2.83 (36.2) |
| Tweed | | 5.78 | 0.24 (4.1) | 3.46 (59.9) | 0.13 (2.2) | 1.95 (33.8) |
| SW Scotland | | 10.74 | 0.29 (2.7) | 6.51 (60.6) | 0.40 (3.7) | 3.54 (33.0) |
| Cairngorms | | 3.85 | 0.18 (4.7) | 2.52 (65.5) | 0.16 (4.1) | 0.99 (25.7) |
| Highlands | | 3.83 | 0.27 (7.0) | 1.37 (35.8) | 0.20 (5.3) | 1.99 (51.9) |
| **Nonstorm Flux** | | | | | | |
| SW England | 4 | 10.24 | 0.50 (4.9) | 7.60 (74.2) | 0.30 (2.9) | 1.84 (18.0) |
| S Wales | 5 | 13.55 | 0.39 (2.9) | 11.53 (85.1) | 0.13 (0.9) | 1.50 (11.1) |
| N Wales | 8 | 14.00 | 0.33 (2.4) | 11.77 (84.1) | 0.25 (1.8) | 1.64 (11.7) |
| Pennines | 6 | 4.64 | 0.12 (2.6) | 2.39 (51.5) | 0.16 (3.5) | 1.97 (42.4) |
| Tweed | 3 | 5.35 | 0.10 (1.9) | 3.60 (67.3) | 0.12 (2.2) | 1.53 (28.6) |
| SW Scotland | 10 | 10.79 | 0.26 (2.4) | 6.99 (64.8) | 0.37 (3.4) | 3.17 (29.4) |
| Cairngorms | 4 | 3.80 | 0.19 (5.0) | 2.52 (66.3) | 0.16 (4.2) | 0.93 (24.5) |
| Highlands | 4 | 3.90 | 0.27 (7.0) | 1.35 (34.5) | 0.19 (4.9) | 2.09 (53.6) |

*Note:* Values in parentheses indicate the percentage contribution each N fraction makes to the total N flux.

area, and percentage cover of different vegetation types was examined by correlation analysis using the annual fluxes for the 52 catchments. $NO_3$ fluxes displayed a significantly positive correlation with percentage cover of improved grass, percentage cover of well-drained soils, and N deposition, and negative relationships with percentage peat cover and percentage cover of blanket bog vegetation (Table 4). Although the DON flux showed a positive correlation with the percentage of peat cover, it showed a stronger relationship with the proportion of poorly draining soils. Fluxes of both PN and $NH_4$ displayed positive correlations with amount of improved grass in the catchment (Table 4).

### DISCUSSION

As the majority of river- and stream-water sampling programs in Britain only analyse for dissolved inorganic forms of N, there is a paucity of information on TN and its DON and PN components. Therefore, the results from this study provide unique information on the range, magnitude, and spatial distribution of DON and TN concentrations and fluxes in upland streams of Britain. The TN content of streams varied significantly between the upland regions of Britain. $NO_3$ accounted for most of the variability in TN; largest concentrations and fluxes were observed in northern and southern Wales and smallest concentrations and fluxes in the Highland and Cairngorm regions in northern Scotland. While this variation in $NO_3$ reflected the pattern of N deposition in Britain, it also reflected variations in land management and the predominant soil type between the upland regions; larger fluxes were associated with well-drained soils and improved grassland. In contrast, DON fluxes were larger in regions with extensive areas of poorly draining soils such as peat and gleys, although the variation in concentrations and fluxes of DON between regions was smaller than for $NO_3$. While fluxes of $NO_3$ observed in this study were considerably larger than those observed in streams draining forested catchments in Sweden and Finland[5], and New England[12], fluxes of $NH_4$ and DON were very similar. In Sweden and Finland, DON fluxes ranged between 1.1 and 4.4 kg $ha^{-1}$ $year^{-1}$, with a median of 1.8 kg $ha^{-1}$ $year^{-1}$, while in New England DON fluxes ranged between 0.9 and 2.4 kg $ha^{-1}$ $year^{-1}$, with a median of 1.2 kg $ha^{-1}$ $year^{-1}$. In all studies, fluxes of $NH_4$ were small (<0.5 kg $ha^{-1}$ $year^{-1}$) and lower than those of $NO_3$ and DON.

The results from this study also demonstrate that N dynamics within upland catchments dominated by poorly drained soils such as peat differ from those in catchments dominated by freely draining mineral soils. In freely draining podzols and brown forest soils, DON is more likely to be readily transformed to $NO_3$ than in the more acidic, anaerobic conditions typically found in peat. Therefore, the relative contribution of DON to TN is likely to be larger in streams draining catchments dominated by poorly draining soils than in streams draining catchments dominated by well-drained soils such as podzols.

While substantial information exists on the riverine transport of dissolved inorganic N fluxes to estuaries and oceans in the U.K.[13], little consideration has been given to organic forms of N which make up the remainder of the N flux. In addition, few studies have assessed the contribution that fluxes of inorganic

**TABLE 4**
**Correlation Coefficients Calculated Between Annual PN, $NO_3$, $NH_4$, and DON Fluxes and Catchment Characteristics for the 54 Study Catchments**

| Catchment Characteristic | PN | $NO_3$ | $NH_4$ | DON |
|---|---|---|---|---|
| N deposition | –0.35* | 0.59*** | NS | 0.28* |
| % Peat | NS | –0.41** | NS | 0.37** |
| % Poorly drained soils | NS | –0.32* | NS | 0.44** |
| % Well-drained soils | 0.30* | 0.50** | NS | –0.28* |
| % Blanket bog vegetation | NS | –0.52** | NS | NS |
| % Improved grass | 0.32* | 0.70*** | 0.28* | NS |
| % Forestry | NS | NS | NS | NS |

*Note:* Significant at the $^{*}p < 0.05$, $^{**}p < 0.01$, and $^{***}p < 0.001$ levels, respectively; NS = not significant.

and organic N from headwater streams make to the total riverine flux. By comparing results from this study with inorganic N loads calculated for U.K. rivers draining to the North Sea[13], it can be seen that as much as a third of the total inorganic N flux transported by rivers to estuaries originates from upland streams. This study has also demonstrated that the DON fraction can represent between 11 and 52% of the total N transported by streams from upland catchments in Britain, with the contribution increasing from southern to northern upland regions. Other studies[14,15] have observed that largest concentrations of DON and smallest concentrations of $NO_3$ occur during the summer, when river flows are small and conditions favour algae growth. The potential availability of DON is therefore very important in these oligotrophic headwaters.

Within the U.K., the bioavailability of DON or its potential role in eutrophication has not been assessed adequately within freshwater systems. There is some evidence that the N from certain low molecular weight compounds can be taken up directly by aquatic bacteria, and conversion of DON to inorganic forms of N can also occur via, for example, photochemical degradation[16]. Although this results in little change in the total N loading in the stream, any transformations that alter the availability of N have an important consequence for water quality and, in particular, eutrophication. There is considerably more information on the bioavailability of DON within estuarine and coastal waters, as N is generally considered to be the nutrient limiting primary production in these systems. For example, 40–80% of the bulk DON in the Delaware and Hudson Rivers was found to be available for bacterial utilisation in their estuaries[17]. The potential availability of DON is particularly important in the interpretation and usefulness of N:P ratios, especially in oligotrophic headwaters which tend towards N limitation during the summer and where DON can be the dominant form of N present[15].

### ACKNOWLEDGEMENTS

The Natural Environment Research Council funded this research (grants GT5/96/2/FS and GST/02/2686). Stream-water samples were collected by the Centre for Ecology and Hydrology, the Environment Agency of England and Wales, and the Scottish Environment Protection Agency; thanks to all the staff involved for their help. Thanks also to Lucy and Henry Chapman for collecting water samples in southwestern England and Yvonne Cook for help with the chemical analysis.

| | | |
|---|---|---|
| Received: | June | 27, 2001 |
| Revised: | September | 18, 2001 |
| Accepted: | October | 10, 2001 |
| Published: | November | 20, 2001 |

### BIOSKETCHES

**Pippa Chapman** is a lecturer in physical geography at the University of Leeds, U.K. Her research interests are concerned with the impacts of atmospheric deposition and land use management on nutrient cycling, particularly nitrogen and phosphorus, in soil and surface waters.

**Tony Edwards** is a senior researcher at the Macaulay Institute, Aberdeen, U.K., which specializes in the science of land use. His research interests focus around the processes that control the behaviour, movement, and fate of nutrients from terrestrial to aquatic systems.
